# Health Aphorisms

**Published:** 1883-11

**Authors:** 


### Health Aphorisms.

Dr. Frank H. Hamilton has formulated the following solid chunks of wisdom:

The lives of most men are in their own hands, and as a rule the just verdict after death would be *felo de se.*

Light gives a bronzed or tan color to the skin; but where it uproots the lily it plants the rose.

Mould and decaying vegetables in a cellar weave shrouds for the upper chambers.

A change of air is less valuable than a change of scene. The air is changed every time the direction of the wind is changed.

Calisthenics may be very genteel, and romping very ungenteel, but one is the shadow, the other the substance, of healthful exercise.

Blessed be he who invented sleep; but thrice blessed the man who will invent a cure for thinking.

Milk drawn from a woman who sits indoors and drinks whisky and beer is certainly as unwholesome as milk from a distillery-fed cow.

Dirt, debauchery, disease, and death are successive links in the same chain.—*Medical Age.*

